# Life stress as a determinant of emotional well-being: development and validation of a Spanish-Language Checklist of Stressful Life Events

**DOI:** 10.1080/21642850.2014.897624

**Published:** 2014-04-04

**Authors:** Roxanna Morote Rios, Odin Hjemdal, Patricia Martinez Uribe, Jozef Corveleyn

**Affiliations:** ^a^ Department of Psychology, University of Leuven, Tiensestraat 102, box 3722, Leuven3000, Belgium; ^b^ Department of Psychology, Norwegian University of Science and Technology, Dragvoll Edvard Bulls veg 1, Trondheim7491, Norway; ^c^ Department of Psychology, Catholic University of Peru, Av. Universitaria 1801, San Miguel, Lima32, Peru

**Keywords:** stressful life events, checklist, validation, psychopathology, HSCL-25

## Abstract

*Objectives*: To develop a screening instrument for investigating the prevalence and impact of stressful life events in Spanish-speaking Peruvian adults. *Background*: Researchers have demonstrated the causal connection between life stress and psychosocial and physical complaints. The need for contextually relevant and updated instruments has been also addressed. *Methods*: A sequential exploratory design combined qualitative and quantitative information from two studies: first, the content validity of 20 severe stressors (*N* = 46); then, a criterion-related validity process with affective symptoms as criteria (Hopkins Symptom Checklist (HSCL-25), *N* = 844). *Results*: 93% of the participants reported one to eight life events (*X* = 3.93, Mdn = 3, SD = 7.77). Events increase significantly until 60 years of age (Mdn = 6). Adults born in inland regions (Mdn = 4) or with secondary or technical education (Mdn = 5) reported significantly more stressors than participants born in Lima or with higher education. There are no differences by gender. Four-step hierarchical models showed that life stress is the best unique predictor (*β*) of HSCL anxiety, depression and general distress (*p* < .001). Age and gender are significant for the three criteria (*p* < .01, *p* < .001); lower education and unemployment are significant unique predictors of general distress and depression (*p* < .01; *p* < .05). Previously, the two-factor structure of the HSCL-25 was verified (Satorra–Bentler chi-square, root-mean-square error of approximation = 0.059; standardized root-mean-square residual = 0.055). *Conclusion*: The Spanish-Language Checklist of Stressful Life Events is a valid instrument to identify adults with significant levels of life stress and possible risk for mental and physical health (clinical utility).

## Literature review

### Introduction

Despite the relevance of stressful events in health and well-being, there is not a valid instrument to investigate their prevalence and impact on Spanish-speaking populations of Latin America. This research aims at developing a screening instrument to evaluate the occurrence of severe stressful life events and provide initial evidence of its clinical utility. With a sequential exploratory strategy, the two studies presented here account for culture and diversity in a non-Western context. They integrate qualitative and quantitative information coming obtained from two independent samples (Creswell, 2003; Yin, 2006).

This paper is divided into four sections and one appendix with the protocol of the Spanish-Language Checklist of Stressful Life Events (SL-SLE).^1^ The first section presents a brief overview of current research on stressful events as determinant of health, particularly in multi-cultural and developing countries. Special attention is paid to the social readjustment model of Holmes and Rahe (1967) because its rationale and items are used to develop a checklist of life stressors for the Peruvian context. The second and third sections correspond to Studies 1 and 2 (i.e. content and psychometric validities); they include methods and results subsections. Additionally, Study 1 describes the rational identification of items, while Study 2 presents the confirmatory factor analysis (CFA) of the criteria used to validate the SL-SLE (Hopkins Symptom Checklist (HSCL-25); Derogatis, Lipman, Rickels, Uhlenhuth, & Covi, 1974). The fourth section discusses the findings and further research with the SL-SLE.

### Stressful life events

The concept of stress involves the environment, an organism, time and outcomes. Each component adds on the possible variability of the stress experience, thus research emphasizes on different aspects according to its objectives (Monroe, 2008). Research on stressful events focuses on natural contexts, appeals to recollections and examines the relations between life events and the psychobiological mechanisms activated, or the possible negative outcomes.

In the last decades, research on stressful life events has shown its relevance and challenges. Firstly, challenges rise from the fissure between a hypothesized objective environmental stress and the subjective appraisals that precede the response. Complex cognitive appraisals focus on how to overcome, prevent or accept the stressful situation (Folkman & Lazarus, 1986; Lazarus & Folkman, 1987). Therefore, individual variability may question a shared criterion to evaluate and compare life stressors. Secondly, confounding is a sensitive issue in the measurement of stress and psychopathology (Lazarus, DeLongis, Folkman, & Gruen, 1985; Luthar & Zigler, 1991). While the last point has been systematically controlled (Paykel, 2001), different research methodologies have dealt with challenges of individual variability and cognitive appraisals.

Interestingly the two most used approaches depart from a shared principle: the cognitive evaluation of a factual stressor will determine to a great extent its consequences. On the one hand, the work of Horowitz, Wilner, and Alvarez (1979) inspired the development of sophisticated methods to assess the perceived stress of any event. On the other hand, the social readjustment model of Holmes and Rahe (1967) provided a method of life stress quantification and established fixed parameters to rate and compare 43 severe life stressors. The authors asked a group of adults (acting like judges) to rate life events according to the amount of readjustment needed to go back to previous homeostasis. An event might be subjectively experienced as positive or negative (controllable or uncontrollable, unexpected or expected), but the comparison and rating process was based on the evaluation of resources needed to face it (readjustment).^2^

Although the use of differential weights for stressors has to be cautious (Skinner & Lei, 1980), the model Social Readjustment Rating Scale (SRRS) shows that it is possible to establish certain standards of life stress comparison. Today the scale is used widely and its predictive validity is demonstrated with regard to specific psychopathologies (Woods, Racine, & Klump, 2010) and physical diseases (Mujakovic et al., 2009). After more than three decades of use and revisions, the SRRS is a commonly used method in life stress research (Hobson & Delunas, 2001; Monroe, 2008; Paykel, 2001).

### Life events research: psychopathology, risk and vulnerabilities

There is well-documented evidence connecting stressful life events with poor psychosocial adjustment and physical distress, either as recent life events or as cumulative events along life (Foster, 2011; Hammen, 2005; Paykel, 2001; Shonkoff & Garner, 2012). Moreover, Scully, Tosi, and Banning (2000) rejected empirically the hypothesis that mainly undesirable and uncontrolled events are at the base of this association. Among others, stressful life events have been associated with anxiety, post-traumatic stress disorder (PTSD), depression, suicide, aggression, addictions, bulimia and psychosis in children, adolescents or adults.

Occasionally, stressful life events are used as a measure of environmental risk. However, researchers are aware that certain environmental risks are stable social or cultural conditions. Epps and Jackson (1993) summarize risk factors as stable conditions in four domains: family, socio-political, cultural and economic contexts. In addition, stressful events have to be differentiated from vulnerabilities. Today, vulnerabilities are mainly studied as biological conditions (genes, hormones and neurological elements) or as early traumatic experience – related to attachment and first relations in developmental research (Masten & Narayan, 2012; Shonkoff & Garner, 2012). In both cases, vulnerabilities are moderators of the impact of life events on well-being.

The occurrence of specific events (e.g. sexual violence) or the accumulation of stressful events is used as a predictor of psychopathology; and usually, their impact is investigated in short periods of time (weeks or months after the event). There are not standard rules for the time elapsed or for the number of severe life stressors to predict physic or psychological outcomes. However, research has demonstrated that the increase of severe life stressors leads to the presence of symptoms. Risch et al. (2009) carried out a meta-analysis of 14 studies where the increment of life events (1–3 or more) predicts depression in adults. Caspi et al. (2003) found similar results for young adults (until 26 years old) and reported 1–3 stressful life events in 86% of a national sample.

The number and characteristic of the life stressors change considerably in multi-trauma or complex trauma research. Here, methods and designs account for circumstances such as war, asylum, force migration (Terheggen, Stroebe, & Kleber, 2001) or environmental disasters (Masten & Narayan, 2012) where the number of life stressors is notably higher. For instance, in war context people suffer mostly up to 11 severe events along their life, although a small group reported up to 27 events (Neuner et al., 2004). In Mexico, Guatemalan refugees reported 8 life stressors in average, and a range of 1–19 events along life (Sabin, Lopes, Nackerud, Kaiser, & Varese, 2003). Additional challenges are posed by research on trans-generational and cumulative risk factors – for instance, studies of abuse and maltreatment in children (Knutson, 1995) or dysfunctional patterns of attachment (Geenen & Corveleyn, 2013). The accumulation of stressors is a bigger threat to cognitive, affective, physical or social well-being (Moen & Erickson, 1995), and it poses additional issues to conventional psychological diagnosis (e.g. PTSD) and treatment (Courtois, 2004).

### Poverty and diversity in the study of stressful life events

There is a lack of research about life stress and symptoms in Latin America, and strong criticisms to the unrevised use of life stress instruments and diagnosis criteria in other non-Western societies (Eyton & Neuwirth, 1984; Gonzales de Rivera y Revuelta & Morera, 1983).

In developing countries or impoverished communities, the study of life stress has to account for social exclusions. Children and adolescents exposed to poverty face deficiencies in health (malnutrition and health services) and education (quality of schooling), lack of stimulation, poor maternal health, fragile connections to home and school, child labor, religious, ritual or military services and parents underemployment (Garmezy, 1993; Grigorenko et al., 2007; Hernandez, 2002; Kotch et al., 1995). Community violence is a complex stressor in contexts of urban poverty. Frequently, it is studied as crime (witnessed and experienced victimization), social insecurity, unemployment, single parenting, lack of infrastructure and services (O'Donnell, Schwab-Stone, & Muyeed, 2002). Here, the challenge for life events research is to differentiate them from demographic characteristics – because of progressive adaptation and possible diffuse symptoms (Rosenthal & Wilson, 2003).

Diversity has broadened the study of life stress and well-being in psychology (Frable, 1997; Stewart & McDermott, 2004). There is an increasing tendency to include gender, ethnicity, education, and social and civil status in life stress research (Bruner et al., 1994; Hobson & Delunas, 2001; Reyes & Acuña, 2008). In Mexican samples, Acuña (2012) and Bruner et al. (1994) found that women, undergraduate students, single participants and those with basic education assigned higher scores to the life events, while Hobson, Kamen, and Szostek (1998) found differences in the evaluation of the stressors by gender and income in the USA. Ethnicity influences both appraisals and frequency of life events: minorities (African, Hispanic or Native Americans) have more in common than Caucasian middle-class groups of the USA due to migration, living conditions, family and community ties (Pine, Padilla, & Maldonado, 1985). Moreover, ethnicity connects to race-related traumatic experiences that act not as differentiated life-threatening events. Racial discrimination within the family is a strong element of internal vulnerability (Sorsoli, 2007) and a trans-generational shared experience (Tummala-Narra, 2007). However, commonly, racism is not considered a traumatic experience or a stressful life event and its consequences in mental health are underestimated.

Sex difference is a profuse field of study in psychology, but the study of gender as a critical category to understand sex-related differences is scarce (Stewart & McDermott, 2004). Broad reviews conclude that there are specific risks, life events and vulnerabilities that lead to negative outcomes for women and men. In the first case, research found consistent evidence connecting stressful events to affective disorders (mainly depression; Hammen, 2005), while in the second case, studies have focused on suicide and poor health (Foster, 2011; Joiner, Brown, & Wingate, 2005). The higher prevalence of depression in women is the most studied sex difference; however, sex-related biological factors cannot account for it, and yet, the meaning of life events are under-explored. For instance, marital status could be risk or protective factor for women depending on cultural values; life events affecting emotional ties have stronger impact on women (Bebbington, 1998; Bebbington et al., 1998). Sex-differentiated roles take in hierarchical status, values, attributions, satisfaction and retributions, thus women's social roles may act as specific affective and social stressors or protection.

In summary, several decades of research has left a main task: to develop valid instruments able to collect retrospective information (memory and cognitive bias) and to account for the stressors' relevance in their contexts. Research has to turn its focus from dysfunctional outcomes to the modifying factors that break the causative chain between life stress and emotional and physical distress (Paykel, 2001). Therefore, the main goal of the following studies is to develop and to validate a screening instrument to evaluate the occurrence and impact of stressful life events in Peru.

## Study 1: development and content validity of the SL-SLE

The aim of Study 1 is to develop a short list of contextually relevant and severe stressful life events for Peruvian adults. The study uses a multi-method approach with expert sampling (Haynes, Richard, & Kubany, 1995).

### Methods

#### Rational selection of items, survey and group interview

First, 28 (of 43) items of the SRRS were selected based on 6 studies: the original SRRS research (Holmes & Rahe, 1967) and 3 revisions, the update of items and national norms (Hobson & Delunas, 2001; Hobson et al., 1998) and an empirical study to address content criticisms (Scully et al., 2000). Because of their misrepresentation of women and ethnic minorities, two studies with Spanish-speaking groups (Mexico) were included: Acuña (2012) and Bruner et al. (1994). Here, subgroups are compared by gender and socio-economic status (SES) using the original items (translated and validated).

There are clear differences in the 20 first positions of the original list of Holmes and Rahe (1967) and the updated version of Hobson et al. (1998). Only original items 1 (‘Death of a spouse’) and 4 (‘Jail term’) kept the same position; items originally rated as 7, 10, 12 15, 18 and 20 changed 23–39 positions. Besides, studies conducted with the original items after 33 and 27 years in the USA (Scully et al., 2000) and Mexico (Bruner et al., 1994) showed important changes in the rating positions.

About the contents, the updating studies of Hobson and Delunas (2001) and Hobson et al. (1998) provide essential information to adapt the items. Firstly, they included 5 new items in the first 20 positions: ‘being a victim of crime’, ‘being the victim of police brutality’, ‘infidelity’, ‘experiencing domestic violence/sexual abuse’ and ‘surviving a disaster’ (rated as 8, 9, 10, 11 and 16 in the new national sample). Secondly, two original items (13, ‘sex difficulties’ and 19, ‘change in number of arguments with spouse’) were replaced by items about family, parenting and sexuality. Thirdly, two original items related to work (15, ‘business readjustment’ and 18, ‘change to different line of work’) were replaced by precise items and were rated in less relevant positions (33, ‘changing employers/careers’; 43, ‘changing positions –transfer, promotion’; 45, ‘changing work responsibilities’). Other changes are the following: original items 3 and 9 (‘separation’ and ‘reconciliation’) were merged and rated in the 12th position; original item 4 (‘jail term’) was broadened to include ‘other institutions’; original item 16 makes explicit the kind of financial ‘change’ (‘problems/difficulties’, rated now 14th), while the new version of item 20 does not specify the amount of money (now rated 47^th^, ‘home mortage’). Once selected the 28 items, the survey and group interview were conducted.

#### Participants

Forty-six Peruvian adults answered a survey and a group interview. They were sorted in 2 groups of women (15 of middle and 11 of low SES) and 2 groups of men (13 of middle and 7 of low SES). Participants of low SES are volunteers of a subsidized non-formal education program for adults living in inland regions. Participants of middle SES are residents of Lima and have pursued professional private education. Participants are 25–60 years old (*M* = 41.2; SD = 9.2); they are born in Lima (*n* = 25) or in regions of Peru (*n* = 21). They have mainly bachelor or postgraduate education (*n* = 25, 77.80%), but 22.2% of the group (*n* = 10) has secondary or technical education.

#### Materials

The survey was designed (1) to rate the relevance of 28 life stressors (three points scale), (2) to assess its accuracy and readability (dichotomous: yes/no) and (3) to suggest new items or modifications. The instrument also collected socio-demographic information. Participants were asked to act as a ‘group of experts’ in order to evaluate the list of life events as significant (or not) according to their experiences or the experiences of others. Instructions included the definition of a ‘relevant stressful event of adult life’ as an ‘event (positive or negative) of great importance in adult life, in which people must use significant amounts of energy, time or resources to adapt to it or to overcome it’.

The group interview was designed as a collective and visual evaluation of the stressors. Based on their individual answers to the survey, participants had to locate the events – written in A4 paper – in a ‘line of severity’ drawn in a blackboard. They had to find certain consensus, discuss and explain their appraisals. The evaluator followed an interview guide.

### Data analysis

Together, qualitative and quantitative information outline the content validity process of the SL-SLE (Creswell, 2003; Haynes et al., 1995).

Quantitative data (survey): Inter-rater reliability (Cronbach's *α*) and cut-off values were calculated for the total group and each subgroup was analyzed (by gender and SES). Cut-off values (means plus one standard error) were used to identify the most relevant stressors (up to 28 selected life events) in the total group and each subgroup. Analyses were done with SPSS (IBM version 22).

Qualitative information (survey's open-ended question and group interviews): It was used to evaluate the linguistic accuracy of the items and to suggest modifications or additional stressors. Group interviews also provided information regarding the appraisals of the stressors. The four group interviews were tape-recorded and transcribed. Coding and analysis was supported by NVivo (QSR version 10). The unit of analyses was themes marked in words, sentences or groups of sentences (Esin, 2011). The analysis will inform the most severe life stressors and in-depth understanding of their impact on adult's lives.

### Results

In average, the groups used 16.5 minutes to answer the survey and 95 minutes in the group interview. The quantitative rating process of the 28 life stressors reached an inter-rater reliability of 0.80 (standardized Cronbach's *α*) for the complete group (*N* = 46) and within-groups from *α* = .72 to .86. Table 1 shows the 18 items rated as highly relevant in the total group (*N* = 46) and four subgroups. The total group pointed out 15 life events as highly stressful (cut-off value for the total group = 2.49). Men agreed on 12 life events, while women and groups divided by SES agreed on 13 life events. Three items rated above a subgroup's cut-off point were included in the final list: ‘Death of close friend’ (included by women), ‘Reconciliation with spouse or partner’ (included by men) and ‘Obtaining a major loan or home mortgage’ (included by the low SES participants). Ten less relevant events were not included in the final list.^3^

**Table 1.** Study 1: rank-ordered life events, means of severity and cut-off values per groups (*N* = 46).

Stressful life events	Total		Women		Men		Low SES		Middle SES	
	Order	*X*	Order	*X*	Order	*X*	Order	*X*	Order	*X*
Death of spouse/mate or partner	1	2.98	1	2.96	1	3.00	1	2.94	1	3.00
Detention in jail or other institution	2	2.91	2	2.88	2	2.95	2	2.89	2	2.93
Surviving a disaster (fire, flood, earthquake, etc.)	3	2.80	4	2.80	4	2.79	4	2.82	4	2.78
Death of close family member	4	2.78	3	2.85	7	2.70	3	2.83	5	2.75
Major injury/illness to self	5	2.76	5	2.77	6	2.75	9	2.61	3	2.86
Important changes in financial state	6	2.65	7	2.65	10	2.65	7	2.67	8	2.64
Separation of spouse or partner	7	2.65	14	2.54	3	2.80	11	2.56	6	2.71
Being fired	8	2.61	8	2.58	9	2.65	8	2.61	9	2.61
Being a victim of crime (assault, robbery, rape, etc.)	9	2.59	16^a^	2.46	5	2.75	5	2.72	14^a^	2.50
Being involved in auto accident	10	2.58	6	2.65	16^a^	2.47	6	2.72	15^a^	2.48
Getting married	11	2.54	11	2.58	13^a^	2.50	17^a^	2.44	10	2.61
Gaining a new family member (birth, adoption, grandparents)	12	2.54	13	2.54	12	2.55	13^a^	2.50	11	2.57
Trying to modify addictive behavior of self (smoking, alcohol, drugs, etc.)	13	2.54	9	2.58	14^a^	2.50	15^a^	2.50	12	2.57
Divorce	14	2.52	15^a^	2.46	11	2.60	19^a^	2.33	7	2.64
Pregnancy	15^b^	2.52	10	2.58	17^a^	2.45	14^a^	2.50	13^b^	2.54
Death of close friend	16^a^	2.46	12^b^	2.54	19^a^	2.35	16^a^	2.44	16^a^	2.46
Reconciliation with spouse or partner	17^a^	2.44	21^a^	2.24	8^b^	2.70	12	2.53	18^a^	2.39
Obtaining a major loan or home mortgage	19^a^	2.35	22^a^	2.23	15^a^	2.50	10^b^	2.56	23^a^	2.21
Mean/SE mean	2.45/0.04		2.44/0.06		2.46/0.07		2.44/0.07		2.46/0.06	
Cut-off value		2.49		2.50		2.53		2.51		2.51

Note: Women *n *= 26; men *n *= 20; low SES *n* = 18 and middle SES *n* = 28.

^a^Item rated below group's cut-off value.

^b^First item rated equal or above group's cut-off value.

Qualitative information (survey's open-ended questions and group interviews) increased the contextual validity of the items (changes in writing style and contents). Participants said that, ‘ … auto accident’ must precise the severity of the incident; ‘Death of close family member’ must clarify that it is about ‘parents, children or siblings’. Participants considered mandatory to eliminate ‘robbery’ of item 9, because it is not comparable with a violent assault or rape. Finally, to equate with other items and to avoid gender bias, the item ‘Getting married’ was phrased as ‘Getting married/living together’.

The groups agreed to include two additional stressors: ‘Conflicts or violence in the family’ and ‘Not to get justice from the state’. The first one built in contents such as forced migration or separation from children or elderly relatives because of migration, money or heir conflicts, maltreatment of children and wife, infidelity, ‘interference of relatives’ in the family affairs, children start living independently and ‘psychological problems’ within the family. The stressor ‘Not to get justice from the state’ included contents such as inefficient, bad (or ‘atrocious’) laws of the country, bad judges, denied access to higher education, social conflicts and social insecurity. Table 2 exemplifies the subjective evaluations of the 10 life events rated as highly severe in the group interviews.

**Table 2.** Study 1: evaluations of relevant and highly stressful life events.

(1) Death of spouse/mate or partner	
	I have seen many people, when you lose your husband or wife, it is very difficult to get use to that, it's like half of you will die, that's what they say … I have close friends, it has been 5 or 6 years and they miss their husband or wife (Man, low SES)
(2) Detention in jail or other institution	
	Horrible, trauma. Not just for you, your children, family, everything around you (Woman, middle SES)
	Your life depends on your decisions, if you loss that, they lock you, you are under somebody's command, an institution that decides for you … What is life if you do not decide for yourself? (Woman, middle SES)
(3) Surviving a disaster (fire, flood, earthquake, etc.)	
	It hits pretty much, suddenly you have nothing, you don't have anything to feed your children, no water, you run, you cannot find anything, it's a very desperate situation, unique (Woman, low SES)
(4) Death of close family member	
	Nothing can substitute a loved one, time buffers the impact but there is always something missing, when you remember you feel broken, it is the love (Woman, Low SES)
(5) Major injury/illness to self	
	You have your dreams, your life projects; I will never think that something like that may happened to me (Woman, low SES)
	Everything is worse for your family, the people around you … maybe you can accept it, the stress is for your children, your husband, your family, that's what would hurts me (Woman, middle SES)
(6) Important changes in financial state	
	I have felt that, I lost half of my salary and I didn't know what to do, is stressful (Man, low SES)
(7) Separation of spouse or partner	
	I have seen many women who have suffered, who commit suicide, because they can say we must be prepared … but we do not know … You cannot be prepared for a sudden change, we are going through other problems (Woman, low SES)
(8) Being fired	
	If you have family, until you get another job, who will support financially the household? when you have a wife, children who are studying, imagine they will ask you money, it's frustrating (Woman, low SES)
	You wonder why, what happened, what did you wrong … I've seen people faint, cry, not understanding why (Man, low SES)
(9) Being a victim of crime (assault, robbery, rape, etc.)	
	The issue of violence, being a victim of a violent act, it is much harder for men because you never foresee to be in that situation, you are more vulnerable (Man, middle SES)
	Two years ago I was assaulted, my tendon, knee and arm had to be plastered, I lost my job, I had many debts … it hurts even today, it was very difficult … I have overcome it little by little (Woman, low SES)
(10) Being involved in auto accident	
	The auto accident is serious psychologically for anyone, even if it is not a sever crash, it stays with you, it happened to me, when I see a car approaching I have that feeling (Woman, middle SES)

When asked about the most stressful events, a participant said ‘everything is about feelings, because everything is related to our families, our close environment’ (Man, low SES). Events referred to the loss of freedom or independence (i.e. jail, illness, accident, finances) elicit appraisals about individual choice, ‘life project’ and personal expectations, although the impact on relatives' life is also mentioned (e.g. events 2, 3, 5 and 8 in Table 2). In fact, the anticipated impact of a personal stressor on own family seems to be experienced as an additional stressor or the worst emotional cost of a life event. In several cases, participants emphasize the impossibility of changing the situation, either just after the event (i.e. disaster, disease, crime) or because it is a stable condition that has to be accepted (i.e. death).

The 10 most relevant stressors are negative life events – although the total list included four positive events (e.g. *Getting married*) and three events that could be considered neutral (e.g. *Obtaining a major loan or home mortgage*). Table 2 shows that participants evaluate the impact of the stressors in terms of the time needed to recover after the events (e.g. events 1, 4, 9 and 10 in Table 2) or the use of inner resources unexpectedly (e.g. events 7 and 8). Clearly, the most stressful events are uncontrolled, unforeseen and they are experienced as changes of lost or harm.

As a result, the protocol of the SL-SLEs was elaborated. It included 20 stressful life events listed in random order. Events are described independently, as a single occurrence, and sex bias was eliminated. Physic or psychological symptoms and personal changes that could be related to previous stressors were not included. The list contains events about family, work and social life; they might be considered either positive or undesirable, as well as controllable or uncontrollable. It is expected that they reflect the experience of diverse segments of Peruvian multi-cultural society. Instructions asked to mark the events experienced along life. The final version of the instrument is in the appendix.

## Study 2, clinical utility: criteria-related validation process

The aim of Study 2 is to get a valid instrument to evaluate the prevalence and impact of stressful life events in Peruvian adults. The concurrent validity of the SL-SLE is established with psychological symptoms (HSCL-25) as related criterion (Cramer & Howitt, 2004).

### Methods

#### Participants

A community sample of 844 adult residents of Lima (older than 18 years) participated in this validation stage. They were contacted through a private university (pre- and postgraduate students), a municipality (social promoters and public servants) and an NGO (volunteers and grassroots leaders). Their individual consent was obtained. Sixty percent of the participants were women; participant's mean age was 29.76 (SD = 11.54) and they were mainly born in Lima (*n* = 541, 65.7%). Their age is distributed between 18 and 25 years old (*n* = 412, 50.6%), 26 and 32 years old (*n* = 152, 18.7%), 33 and 41 years old (*n* = 111, 13.6%), and 42 and 76 years old (*n* = 139, 17.1%). Participants have undergraduate (*n* = 507, 61.40%), postgraduate education (*n* = 222, 26.90%), and secondary or technical education (*n* = 97, 11.70%). Participants study or study and work simultaneously (*n* = 549, 69.60%), 84 are subemployed (temporary or independent jobs, 10.6%), 138 participants have stable jobs (17.50%) and 18 participants are unemployed or housewives (no income, 2.20%).^4^

### Materials

#### Spanish-Language Checklist of Stressful Life Events

The instrument includes 20 positive and negative life stressors (Study 1) and also collects socio-demographic information (gender, age, place of birth, education and employment). Participants were asked to put a check mark next to the events experienced along life. SL-SLE total score is calculated as the unweighted sum of answers to the items (1 = had ever been experienced; 0 = had not). It is expected that higher number of events reflects an increase in life stress (continuous variable, 0–20).

#### Hopkins Symptom Checklist

The 25-item version of the instrument of Derogatis et al. (1974) is a measure of general distress, depression and anxiety symptoms. The pencil-and-paper self-report asks about the presence of symptoms over the last week. Item responses range from ‘not at all’ (1) to ‘extremely’ (4). The increase of the score indicates an intensification of symptoms. Even though there are exceptions (Hermansson, Timpka, & Thyberg, 2003; Ventevogel et al., 2007), a cut-off point of 1.75 is used for screening and/or classification (sensitivity and specificity) in Western countries (Sandanger et al., 1998, 1999; Tinghög & Carstensen, 2010), African, Middle East, Asian countries (Kaaya et al., 2002; Mollica, Wyshak, & Lavelle, 1987; Mouanoutoua & Brown, 1995; Silove et al., 2007), and Latin American countries (Sabin et al., 2003; Tremblay, Pedersen, & Errazuriz, 2009). These studies also show the good internal consistency (standardized Cronbach's *α*) of the HSCL-25 in these settings: generally, *α* > .90 for the total score and *α* > .80 for anxiety and depression.^5^

#### Data analysis

Mplus6 was used to estimate model parameters and run the CFA of the HSCL-25. Descriptive and inferential analyses were performed with SPSS (IBM version 22). Descriptive statistics of each life stressor and meaningful differences between subgroups were explored (non-parametric analyses). Four-step hierarchical regression analyses tested the criterion-related validity of the SL-SLE (concurrent).^6^

There is no evidence of the measurement model of the HSCL-25 in Peru or Latin America, thus its two-factor structure was tested (CFA, anxiety and depression, composed of 10- and 15-factor indicators, respectively). Robust maximum-likelihood estimation (MLM) and the Satorra–Bentler chi-square (SB *χ* ^2^) were used in order to account for the non-normality of the data. Then reliability of the scales was confirmed (Cronbach's *α*).

Modeling with hierarchical regression analyses explored the association between life stress and psychology symptoms. The continuous variables were log transformed, age was mean centered and HSCL-25 outliers were controlled. Three four-step regression models differentiate systematically the effect of gender, age, place of birth (step 1), education (step 2), employment (step 3) and life stress (step 4, SL-SLE) in predicting three-symptom scores (effect indicators): HSCL anxiety, depression and total score. Adjusted *R* ^2^ was compared between participants with an SL-SLE total score up to 8 life events (*N* = 751, three models) and all the participants (*N* = 811, three models).

### Results

#### SL-SLE descriptive information

Table 3 shows the frequencies and percentages of each life stressor in the total group (*N* = 844) and groups by gender and place of birth.

**Table 3.** Study 2: order, frequency and percentage of stressful life events in the total sample (*N* = 844) and subgroups by gender and place of birth.

Life event	Total			Men^a^			Women^b^			Lima^c^			Region^d^		
	Order	*f*	%	Order	*f*	%	Order	*f*	%	Order	*f*	%	Order	*f*	%
Gaining a new family member (birth, adoption, grandparents)	1	404	0.47	1	149	0.37	1	250	0.62	1	258	0.65	2	136	0.34
Changing economic status	2	370	0.43	2	141	0.38	2	224	0.61	2	218	0.60	1	141	0.39
Being a victim of crime (assault, rape)	3	315	0.37	3	129	0.41	4	182	0.58	3	212	0.69	4	95	0.30
Death of close family member (parents, siblings, children)	4	312	0.37	4	125	0.40	3	183	0.59	4	188	0.61	3	118	0.38
Surviving a disaster (fire, flood, earthquake)	5	244	0.28	5	97	0.40	6	144	0.59	5	154	0.64	7	84	0.35
Conflicts or violence in the family^e^	6	234	0.27	8	70	0.30	5	161	0.69	6	144	0.62	6	85	0.37
Getting married	7	198	0.23	7	75	0.38	7	119	0.61	8	96	0.51	5	91	0.48
Death of close friend	8	186	0.22	6	82	0.44	8	102	0.55	7	105	0.57	8	77	0.42
Being fired/laid-off/unemployed	9	160	0.19	6	82	0.51	10	76	0.48	10	86	0.54	9	71	0.45
Separation of spouse/mate	10	137	0.16	10	43	0.31	9	92	0.68	11	75	0.56	10	57	0.43
Attempting to modify addictive behavior of self (smoking, alcohol, drugs)	11	129	0.15	9	60	0.46	11	69	0.53	9	90	0.71	13	36	0.28
Pregnancy	12	117	0.13	17	12	0.10	8	102	0.89	12	56	0.49	10	57	0.50
Not to get justice from the state^e^	13	106	0.12	11	42	0.40	13	61	0.59	14	54	0.51	11	51	0.48
Obtaining a major loan or home mortgage	14	101	0.12	12	34	0.34	12	65	0.65	15	46	0.49	12	47	0.50
Major injury/illness to self	15	83	0.09	14	29	0.34	14	54	0.65	13	55	0.67	15	26	0.32
Reconciliation with spouse/mate	16	77	0.09	13	32	0.41	15	45	0.58	15	46	0.62	14	28	0.37
Experiencing a severe auto accident	17	65	0.07	14	29	0.44	16	36	0.55	16	41	0.66	16	21	0.33
Divorce	18	40	0.04	16	13	0.32	17	27	0.67	17	23	0.57	17	17	0.42
Detention in jail or other institution	19	29	0.03	15	17	0.62	18	10	0.37	18	15	0.53	18	13	0.46
Death of spouse/mate	20	9	0.01	18	1	0.12	19	7	0.87	19	3	0.33	19	6	0.66

^a^ *n* = 333.

^b^ *n* = 501.

^c^ *n* = 541.

^d^ *n* = 283.

^e^Items added by qualitative study of content validity.

The four most frequent events in the total sample are the most frequent events across the subgroups (they change only one position). More variability is observed in the frequency of events reported for those who are not born in Lima: not any of the seven most frequent events in the total group keep the same position in the first generation of migrants in Lima. Table 3 shows also the relevance of the items qualitatively added to the list of stressors (Study 1). They appear ranked in position numbers 6 and 13.

The total score of the SL-SLE ranges from 0 to 16 life events (*N* = 844, *M* = 3.93, Mode = 2, Mdn = 3, SD = 7.77). As expected, it has a non-parametric positively skewed distribution (K–S = 0.153, *p* = .000; S–W = 0.924, *p* = .000): skew of 0.972 (SE = 0.084) and kurtosis of 0.849 (SE = 0.168). Subgroups by gender, age, place of birth and education show similar non-parametric distribution (*p* < .001). Based on the literature review (Courtois, 2004; Neuner et al., 2004; Sabin et al., 2003) and the identification rule of Hoaglin and Iglewicz (1987) (*g*′ = 2.2), a cut-off point was fixed in eight events. As expected, 93% (*n* = 751) of the participants experienced 8 or less events along life, while only 7% of them (*n* = 60) declared 9–16 events.

In order to compare SL-SLE total score between subgroups (Mann–Whitney *U* and Kruskal–Wallis *K*), homoscedasticity was explored (Levene's test with rank transformations; Nordstokke & Zumbo, 2010). Variances are homogeneous in the subgroups defined by gender (*F*(1, 833) = 0.839, *p* = .360), place of birth (*F*(1, 823) = 0.771, *p* = .380) and education (*F*(3, 825) = 1.361, *p* = .254). The variable age has non-homogeneous variances between subgroups (*F*(4, 813) = 2.551, *p* = .038). There are no significant differences in SL-SLE total score by gender (Mdn = 3, *U* = 80874.500, *p* = .452). On the contrary, there are significant differences in participants who were born in inland regions of Peru (Mdn = 4) and in Lima (Mdn = 3) (*U* = 64342.500, *p* < .001, *r* = −0.13, two-tailed).^7^ There are significant differences in SL-SLE total score in the three levels of education (*H* (2) = 59.138, *p* < .001, *r* = 0.072^8^). Pairwise comparisons show differences between categories (*p* < .001): secondary and technical education (Mdn = 5); undergraduate education (Mdn = 3) and postgraduate education (Mdn = 4).

As expected, stressful life events are positively associated with age (Pearson *r* (776) = .41, *p* < .001), thus SL-SLE total score was compared by age groups. The median of SL-SLE is significantly different across age groups (Mood's median test *χ* ^2^ (4, 814) = 122.305, *p* < .01, two-tailed). Pairwise comparisons revealed significant differences between 18–25 (Mdn = 2) and 26–32 years old (Mdn = 4) (*χ* ^2^ (1, 564) = 18.418, *p* < .01, two-tailed); between 26–32 and 33–41 years old (Mdn = 5) (*χ* ^2^ (1, 263) = 9.169, *p* < .01, two-tailed); and between 33–41 and 42–60 years old (Mdn = 6) (*χ* ^2^ (1, 239) = 12.822, *p* < .01, two-tailed). There is no significant difference between adults from 42 to 60 and 61 to 76 years old (Mdn = 6) (*χ* ^2^ (1, 139) = 1.230, *p* < .428, two-tailed).

#### HSCL-25 factor validity

First, participants (30) with more than one missing response in any HSCL item were excluded – Little's missing completly at random test: *χ* ^2^ (504, *N* = 841) = 572,030, *p* = .019. One missing completely at random value was imputed in 40 cases (4.9% of the sample) with a single expectation maximization process (Scheffer, 2002). Then, inter-item correlations were verified in order to proceed with the CFA.

The two-factor model of the HSCL-25 fits the data well in the Peruvian sample. The overall fit indices for the CFA are S–B *χ* ^2^ (274) = 1047.786, S–B *χ* ^2^/df = 3.824, *p* < .001, and scaling correction factor for MLM: 1.431. Following the general criteria of Hu and Bentler (1999), the root-mean-square error of approximation (RMSEA) is below 0.06 (RMSEA = 0.059; 95% IC RMSEA = 0.055–0.063) and the standardized root-mean-square residual (SRMR) is below 0.08 (SRMR = 0.055). RMSEA corrects for parsimony and provides a test against the perfect model, thus it is essential in judging a model with 25 indicators. However, and similarly to results reported by Al-Turkait, Ohaeri, El-Abbasi, and Naguy (2011) in an Arab sample, the criteria for the comparative fit index (CFI) and the Tucker–Lewis Index (TLI) (above 0.90) were not met (CFI = 0.830; TLI = 0.814). The regression weights (factor loadings) are all significantly different from 0 (*p* < .001, two-tailed). The covariance paths between the factors (anxiety and depression) are significantly different from 0 (*p* < .001, two-tailed).

HSCL-25 total scale and subscales (anxiety, 10 items and depression, 15 items) show excellent or good internal consistency (Cronbach's *α*) in the Peruvian sample (George & Mallery, 2003; Nunnally & Bernstein, 1994); HSCL-25 total: *α* = .904, anxiety: *α* = .806 and depression: *α* = .864. HSCL-25 scores were computed as the weighted sum of answers divided by the number of items of each scale. Outliers were controlled for HSCL total score (four cases), anxiety (six cases) and depression (four cases) (Hoaglin & Iglewicz, 1987) prior to explore the basic statistics of the scales. HSCL total (*n* = 807), *M* = 1.49 (SD = 0.36), skew = 1.04 (SE = 0.09) and kurtosis = 0.71 (SE = 0.17); HSCL anxiety (*n* = 805), *M* = 1.49 (SD = 0.36), skew = 0.91 (SE = 0.09) and kurtosis of 0.60 (SE = 0.17); and HSCL depression (*n* = 807), *M* = 1.49 (SD = 0.39), skew = 1.08 (SE = 0.09) and kurtosis = 0.82 (SE = 0.17). In the Peruvian sample, 79.7% of the participants are below the international cut-off point of 1.75 for the total score, 78.9% for anxiety and 79.1% for depression.

#### Modeling SL-SLE

Six four-step hierarchical multiple regression analyses were carried out with HSCL anxiety, depression and total score as dependent variables. Ten independent variables were organized in four steps to distinguish their capacity to account for by the outcomes' variance. The main variable of interest, SL-SLE total score, was introduced at the fourth step to explore its predictive capacity above the others. Independent variables are not highly correlated, with the exception of stressful life events and age. The collinearity statistics (i.e. tolerance and variance inflation factor (VIF)) are all within accepted limits – the lowest levels of tolerance is 0.535, while the highest level of VIF is 1.871.

All the regression models for the dependent variables show significant predictive capacity (adjusted *R* ^2^, with *F* parameters at *p* = .000). Analysis including participants with one to eight stressful life events along life (*n* = 751) showed stronger adjusted *R* ^2^ than models tested with all the participants (*n* = 811).^9^ Thus, the first set of analyses has better predictive capacity^10^ (Table 4).

**Table 4.** Summary of hierarchical regression analyses for variables predicting HSCL total score, anxiety and depression.

	HSCL total^a^				HSCL anxiety^b^				HSCL depression^c^			
Predictor	*β*		Δ*R*^2^	Δ*F*	*β*		Δ*R*^2^	Δ*F*	*β*		Δ*R*^2^	Δ*F*
Step 1: Demographics		0.029		7.822***		0.053		13.959***		0.011		3.685*
Gender	0.118**				0.121**				0.101**			
Born in Lima	−0.021				−0.049				0.008			
Age	−0.165**				−0.180***				−0.135**			
Step 2: Education		0.037	0.013	3.091*		0.056	0.007	1.695		0.023	0.016	3.723*
Secondary	0.095*				0.047				0.112**			
Technical	0.079*				0.063				0.080*			
Undergraduate	0.088				0.091				0.073			
Step 3: Occupation		0.041	0.007	1.791		0.055	0.003	0.686		0.029	0.010	2.415
Student and working students	0.042				0.051				0.029			
Subemployed	−0.019				−0.020				−0.017			
Unemployed and housewives	0.087*				0.041				0.105**			
Step 4: Life stress		0.068	0.028	20.984***		0.072	0.018	13.570***		0.054	0.026	19.405***
Stresful life-events	0.179***				0.144***				0.174***			

Notes: Education and employment are dummy variables with 0: ‘graduated’ and 0: ‘employed’ as the reference group, respectively. Age is centered at its mean. Standardized beta weights (*β*) belong to the fourth step.

^a^ *n* = 696.

^b^ *n* = 695.

^c^ *n* = 696.

*p < .05.

**p < .01.

***p < .001.

The hierarchical multiple regression analysis revealed that each of the four steps contributes to the regression models (ANOVA, *p* < .001). Stressful life events (step 4) increases significantly and consistently the prediction of general symptoms, anxiety and depression (*p* = .000), followed by step 1, demographic characteristics in the prediction of general distress and anxiety (*p* < .000) and depression (*p* < .05). Step 2 (education) significantly increases the prediction of general distress and depression (*p* < .05). The increase of this contribution (adjusted *R* ^2^ and *F* change) is not significant only at step 3 for any criteria (‘occupation’ as a block of variables).

When all the independent variables are compared in step 4 of the regression models (standardized beta weights, *β* in Table 4), stressful life events remain as the most important predictor of psychological symptoms. It explains uniquely 18% of the variation in general distress, 14% of the variation in anxiety and 17% of the variation in depression. Gender and age have a unique predictive capacity of the three dependent variables. The direction of the associations shows that women and younger participants have stronger probabilities to show higher scores in the three-symptom scales. Lower levels of education (secondary and technical) and unemployment (unemployed participants and housewives) are also unique predictors of general distress and depression.

## Discussion

Results show that the SL-SLE is an empirically supported and evidence-based instrument to investigate the prevalence and impact of stressful life events in Peruvian adult population (Holmbeck & Devine, 2009). Although further research should extend this conclusion, the SL-SLE shows satisfactory psychometric characteristics and capacity to identify adults at risk of developing symptoms of anxiety and depression.

In accordance with the literature, the content and psychometric validity processes of the SL-SLE resulted in negative (13), positive (4) and neutral (3) events, as well as uncontrollable (9) and controllable (11) events. These events correspond to diverse domains of adult life (relationships, work, family and community life) and they may activate internal and external resources in the individual. The quantitative study provided initial evidence of risk associated with the number of events (specially for age groups and birth place). However, whether or not a person will go through psychosocial or physical impairments will depend on diverse conditions, such as previous life experiences, psycho-biological characteristics, the accumulation of stressors and mainly, the subjective evaluation of the event and its consequences. The SL-SLE does not assess objective stress; it aims at providing a shared criterion to identify potentially harmful life events for the emotional well-being of adults in Peru.

The relevance of life events related to family and community ties has been studied as a cultural characteristic of Latin American (Hernandez, 2002) and Peruvian (Elsass, 2001) populations. The new items developed for the SL-SLE reveal that family and social contexts are simultaneously a domain of meaningful personal experience and source of stress and vulnerability.

The study provides normative information about Peruvian adults' life stress. These findings can lead us to some conclusions and further paths of research. In accordance with the literature, the number of stressors does not differ for men and women, and there is a significant increase of life events by age (from 3 to 6 events between 18 and 60 years of age). In both cases, patterns of life events may be explored to better understand the influence of gender and age in life stress. Interestingly, the life event ‘change of residence (migration)’ was excluded from the group of highly relevant stressors (content validity study). However, the validation process showed that being a migrant in Lima determines an experience of numerous and stressful life events. Although migration may entail a transition out of poverty, especially for young adults (Crivello, 2009), internal and external migration in Peru is a phenomenon associated with lack of social opportunities. It comprises the departure from contexts of severe exclusion (i.e. services of health or education) to the insertion in contexts of urban poverty. Recently, the differential and severe effect of these stressors has been studied as pre- and post-migration stress and has been empirically connected to mental health symptoms in Peruvian migrants (Lahoz & Forns, 2013). Further research is needed to understand in depth the short- and long-term impact of migration for adults' well-being in Peru or other Latin American contexts.

The exploration of SL-SLE total scores revealed unforeseen results: there is a small group of participants (4.9%) who declare an unusual number of stressors along their lives: from 9 to 16. This amount of stressors is not only labeled as outliers by psychometric procedures (Hoaglin & Iglewicz, 1987) but it is comparable to the number of stressors reported in contexts of war and forced migration (refugees) (Neuner et al., 2004). Contrary to what could be expected, the inclusion of this highly stressed group weakened the predictive power of life stress on psychopathology (hierarchical models with *N* = 811 and Pearson's *r*). These preliminary findings are consistent with current research on multi-traumatized groups. They not only experience unusual amounts of life stressors but also show patterns of complex physical and psychological outcomes. The first ones are mainly connected to cumulative experiences of child abuse, neglect, social violence and disasters, while the outcomes are a challenge for diagnostics and treatment (Courtois, 2004). Further research is needed to clarify the specific characteristics of these participants, their vulnerabilities, possible negative outcomes as well as resources developed to face the repetitive occurrence of severe stressors.

The most important result to emerge from the data is the significant and consistent predictive capacity of stressful events assessed with the SL-SLE – on psychopathology symptoms. Modeling with hierarchical multiple regressions demonstrate its capacity to identify systematic variations of symptoms' scores as a function of distal and complex background characteristics (Bryk & Raudenbush, 1987). Two conditions of the study make these findings specially challenging. Firstly, the SL-SLE did not explore a recent or fixed period of time. Commonly, studies focus on proximate events and connect them to reactive episodes of anxiety (mainly PTSD) or depression (for instance, after a significant lost) (Kessler, 1997; Silove et al., 2007). In this study, cumulative stressors and the uncontrolled time between events and outcomes could obscure their impact on well-being. Secondly, as suggested by Luthar and Zigler (1991), this research does not control the presence and intensity of the dependent variables (psychopathology) by focusing on clinical samples. On the contrary, it kept a community-based approach, looking for a broad and diverse group of participants. This study aimed at developing an accurate instrument for researching in natural contexts, prioritizing the cultural and social diversity of the target population.

In accordance with research in Western and non-Western contexts, education and employment, along with stressful life events, consistently predict dysfunctional responses and greater risk. Clearly, indicators of social disadvantage (lower education, employment insecurity or unemployment) play a key role in psychosocial well-being of individuals or in the accumulation of stressors (migration).

Age and gender are specially challenging for further research. Age is the most consistent and powerful socio-demographic predictor of mental health (anxiety, depression and general distress), thus it is important to investigate youngsters' life conditions and inner resources as a risk factor in the Peruvian context. Women show greater risk of depression and general distress. This is consistent with previous findings reported in the introduction, and it also provides some paths of analysis for gender. Social roles and expectations ascribed to women might be affecting their well-being. Roles such as family or community caregivers – especially for migrant and poor women – single parenting or social leadership could be associated with symptom manifestation (Bebbington et al., 1998; Ventevogel et al., 2007) or subjective distress (Morote, 2011). Distal risk factors – i.e. child neglect/abuse – are associated with women's depression (Bifulco, Brown, Moran, Ball, & Campbell, 1998), and the combination of distal risks – such as maternal loss (Tennant, 1988) and early absence of own mother (Kotch et al., 1995) – with current stressors are major predictors of women's depression. It is important to contextualize women's regulation of emotions because it is not purely individual processes, but culturally constructed patterns of adaptation with different consequences in women's health (Butler, Lee, & Gross, 2007).

Finally, although the results provide evidence of the accuracy of the HSCL-25 scales of anxiety and depression, further research should expand our understanding of somatic symptoms in the expression of emotional distress. In this study, symptoms such as headaches, trembling, faintness, dizziness or weakness, and difficulty falling asleep, showed unexpected patterns of correlations with their scales. Cross-cultural clinical research has shown that some expressions of mental distress are culturally dependent. More precisely, unusual expressions of psychosomatic symptoms have been identified in Middle East (Tinghög & Carstensen, 2010), Asian (Pernice & Brook, 1996; Terheggen et al., 2001) and African countries (Kaaya et al., 2002). In Peru, research has already shown the necessity of specific methods to assess subjective and somatic distress in traumatized Quechua groups – such as worrying memories, headaches, stomach or chest pains, convulsions and general weakness. Traditional psychometric instruments of depression, anxiety or PTSD may find little support in non-Western contexts, while research including culturally relevant outcomes and adapted instruments showed consistent explanatory power (Tremblay et al., 2009).

The sampling method may be considered as a limitation of Study 2. However, as explained, accessibility and diversity were the main criteria to reach the participants, thus several recruiting strategies were used to improve the sample composition. For instance, different institutions (i.e. a university, local and metropolitan municipalities and an NGO) were contacted, and the demographic characteristics of the sample were inspected during the data collection. As a result, group comparisons are possible with the validation sample though generalization has to be cautious.

The impact of severe stressors on the development of symptoms is more obvious when time is limited to 1, 5 or even 10 years. However, the final format of the SL-SLE does not exclude the possibility to restrict the time assessed. This might be a convenient choice in further research, responding to specific objectives or target groups.

Prospective-longitudinal studies would also broaden the clinical utility of the SL-SLE. The capacity of the SL-SLE to predict affective symptoms, PTSD or physical conditions could be tested in series of time points. The increase of stressful events in fixed periods of time is not only related to affective symptoms but their interaction with genetic conditions can be demonstrated (Caspi et al., 2003). The use of the SL-SLE as a screening instrument (to gather information at an early stage of a relevant risk condition) could be used to prevent or to monitor treatments in diverse physic and psychological conditions.

Finally, further research could also include clinical samples or valid diagnostic instruments in order to screen for psychopathology. The promising results of the HSCL-25 CFA allow trustworthy use of the HSCL-25 in Peru.

In conclusion, these findings represent an initial validation of a useful instrument to evaluate the occurrence and impact of relevant life stressors for adults in a Latin American sample. Satisfactory results were obtained to support its capacity to identify adults at psychosocial risk. This study also provides a springboard for the study of life stress in natural contexts and to search for connections with possible negative or positive outcomes. Among others, promising applications of the SL-SLE are the exploration of distinctive patterns of stressors for men, women, migrants, disadvantaged groups and for people with unusual accumulation of stressors along life. The usefulness of the SL-SLE has to be proved in the prevention and management of physical and mental consequences of life stress. The exploration of moderating factors of the impact of stressful life events is a promising area of further research.

## Notes

1 Spanish version: ‘Listado de Eventos de Vida Estresantes en Español – LEVE’.

2 Once established the norms per event (‘Life Change Units’), the authors used the scores in an additive manner to determine life stress of each individual in a fixed period of time (generally in the last year). In Study 1, quantitative rating process of events and the qualitative appraisals of their severity followed the rationale of the model of Holmes and Rahe (1967).

3 The items are ‘illness of close family member’, ‘assuming responsibility for sick or elderly loved one’, ‘change in work hours or conditions’, ‘retirement’, ‘change in number of arguments with spouse’, ‘sex difficulties’, ‘change to different line of work’, ‘no health insurance’, ‘trouble with boss’ and ‘change in living conditions (migration)’.

4 Subsample number of participants for each category does not always add up to 844 due to missing information.

5 The translation into Spanish used was made by the Harvard Program in Refugee Trauma and published by Oficina en México del Alto Comisionado de las Naciones Unidas para los Derechos Humanos (2007).

6 Reliability and dimensionality reduction are inappropriate analyses to validate a checklist of life events (Shalowitz, Berry, Rasinski, & Dannhausen-Brun, 1998; Tremblay et al., 2009).

7 The effect size *r* was calculated by dividing *Z*(−3, 793) by the square root of *N* (824).

8 The effect size *r* was calculated by dividing the chi-square value *H* (2) = 59.138 by *N*−1 (825).

9 Results with *N* = 811: HSCL total score (*n* = 754, , *F*(10, 743)* *=* *6.714 , *p = *.00), anxiety (*n* = 753, , *F*(10, 742) = 6.714, *p = *.00) and depression (*n* = 754, , *F*(10, 743) = 5.022 , *p *= .000).

10 Interestingly, strong direct associations (*r*) were found between the SL-SLE total score and HSCL-25 scales (means) but just until SL-SLE total score reaches eight events (*N* = 751, 93% of the sample). *R* ^2^ of stressful life event (SLE) (0–8) with HSCL total score is 0.484, with anxiety 0.376 and depression 0.411. When included the small group of participants with 9–16 life events (*n* = 59), the association is not significant: *R* ^2^ of SLE (0–16) with HSCL total score is 0.051, with anxiety 0.023 and depression 0.084.
